# Oxygen transport during liquid ventilation: an in vitro study

**DOI:** 10.1038/s41598-022-05105-1

**Published:** 2022-01-24

**Authors:** Katrin Bauer, Thomas Janke, Rüdiger Schwarze

**Affiliations:** 1 Institute of Mechanics and Fluid Dynamics, TU Bergakademie Freiberg, Freiberg, Germany; 2 LaVision, Göttingen, Germany

**Keywords:** Biomedical engineering, Fluid dynamics, Optical sensors

## Abstract

An in vitro experiment on the dissolved oxygen transport during liquid ventilation by means of measuring global oxygen concentration fields is presented within this work. We consider the flow in an idealized four generation model of the human airways in a range of peak Reynolds numbers of $Re = 500$–3400 and Womersley numbers of $\alpha = 3$–5. Fluorescence quenching measurements were employed in order to visualize and quantify the oxygen distribution with high temporal and spatial resolution during the breathing cycle. Measurements with varying tidal volumes and oscillating frequencies reveal short living times of characteristic concentration patterns for all parameter variations. Similarities to typical velocity patterns in similar lung models persist only in early phases during each cycle. Concentration gradients are quickly homogenized by secondary motions within the lung model. A strong dependency of peak oxygen concentration on tidal volume is observed with considerably higher relative concentrations for higher tidal volumes.

## Introduction

Since its first mention in 1962 by Kylstra et al.^1^, Liquid Ventilation (LV) has been proposed as a promising ventilation strategy for various complications occurring in intensive care. Greenspan et al.^2^ suggested the treatment of preterm neonates with a LV protocol. They could show an improvement in lung function and underlined the advantages of the utilized perfluorocarbon (PFC) liquids (high gas solubility, low surface tension, distributes evenly). Furthermore, liquid ventilation has also been proposed to treat adult patients with acute respiratory distress syndrome (ARDS)^3^. But until today, liquid ventilation lacks a proof of performing better than conventional mechanical ventilation (CMV)^4^ for this case. More recent studies suggest to incorporate hypothermic total liquid ventilation (TLV) after cardiac arrest^5–9^. Such a protocol has not only the advantage of providing ventilation for the patient but it can also protect the cardio- and neurosystem by ultra-fast cooling. To do so, a body temperature of around 33 $^\circ$C (mild hypothermia) has to be achieved within the shortest time possible. As Chenouve et al.^5^ showed, cooling by TLV can be three to four times faster in comparison to conventional cooling strategies. From a fluid dynamic perspective, liquid ventilation has just been sparsely investigated. While the results of researches concerning the fluid’s behavior during gas ventilation may be transferable to liquid ventilation strategies based on comparable characteristic numbers (Reynolds number, Womersley number), only a few dedicated studies have been published in order to gain new insights into the processes of liquid ventilation. Some of these studies have been summarized by Halpern et al.^10^. This review mainly covers the transport of liquid plugs in confined channels. Such process may be most important during filling of the lungs. After successful administration of the liquid, the transport mechanisms of dissolved gases play a crucial major role for providing enough fresh oxygen. While a few approaches have been taken of imaging the oxygen distribution within the lungs^11–14^, they are all limited by the achievable spatial resolution and mainly the determination, whether certain regions are preferably supplied with PFC or not. Within the work here presented, we want to introduce an in vitro study on the dissolved oxygen transport and distribution modeling the flow within the conductive airways of the human lung. First experiments have been presented in the past by our group^15,16^. Within these works, we investigated dissolved oxygen concentrations during constant inspiration and expiration and could identify characteristic distribution patterns. In this new work, we investigate the influence of oscillatory flow on the distribution and transport patterns during different phases of the breathing cycle.

## Methods

### Lung model

**Figure 1:**
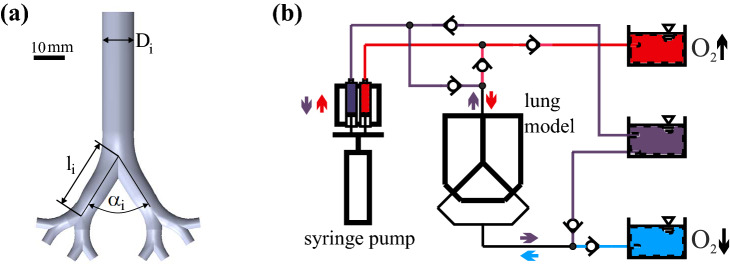
(**a**) Geometry of the lung model, (**b**) sketch of the experimental set-up illustrating the flow paths between the syringe pump and the storage liquid containers. Red colors denote initial high concentration, blue low concentration, purple is a mixed concentration.

The model features four symmetrical airway generations with circular cross sections. The geometry is based on the model presented in^17^ as flow visualization measurements, i.e. particle image velocimetry (PIV) measurements have been carried out in the similar geometry. Starting from the largest channel ($D_1 = 10.48$ mm), it bifurcates three times and the channel’s diameter reduces down to ($D_3 = 3.3$ mm) as minimum diameter (see Fig. 1a). With these dimensions it hence represents an adult lung from approximately the 2nd to the 5th bifurcating generation , featuring a model dead space of about 10 ml . The experimental model is made of polymethyl methacrylate (PMMA) and nine hose connectors are attached, one at the largest channel and eight at the distal ends. All relevant geometrical dimensions are given in Table 1.

**Table 1:** Geometrical characteristics of the simplified lung model.

Model generation *i*	$D_i$ (mm)	$l_i$ (mm)	$\alpha _i$ (°)
0	10.48	47.6	
1	7.86	22.6	64
2	4.98	9.22	66
3	3.3	6.72	68

#### Model limitations

The lung model employed here with flat, symmetric bifurcations features a strong simplification of the real lung. Thus, additional rotations of the Dean vortices, which can be observed in a fully three-dimensional model geometry^18^ , cannot be generated here. Due to the rigid structure any compliances as well as collapsible structures can also not be considered here. However, the geometry is sufficient to cover the main features of convective lung flow and gas transport. The big advantage of planar lung models is the option of a simultaneous optical flow analysis of all mother and daughter branches. The round cross sections and bifurcating branches thus allow the generation of the characteristic Dean vortex patterns, typical flow split-up as well as skewed velocity profiles^19^ . By variation of the breathing parameters, laminar as well as turbulent flow conditions can be covered here. The simplified geometry hence allows a better systematic study of flow induced gas concentration and mixing patterns.

### Experimental set-up

The lung model is fixed horizontally on an optical bench. It is connected to a syringe pump and three liquid storage containers via several flexible hoses. Two different flow paths are needed to supply the model with liquid of high or low oxygen concentration during inspiration and expiration, respectively. This is achieved with Y-connectors and non-return valves (see Fig. 1b). Two of the storage containers are used to supply the model with liquid of initially low and high concentrations, respectively, while the third acts as an overflow tank.

The syringe pump consists of a linear actuator (LinMot) driving four syringes. The linear motor follows a sinusoidal motion with adjustable stroke and frequency. Two of the four syringes are grouped to either contribute to the inspiration or the expiration phase, respectively. While the syringes push liquid with high oxygen concentration (red arrow) through the model during inspiration, liquid of low oxygen concentration (blue arrow) is pulled from a storage container through the eight distal channels during expiration. The determination of the dissolved oxygen concentration fields is carried out for six different tidal volumes ( $V_{\mathrm{t}} = 30, 40, 50, 60, 80, 100$ ml) and three oscillation frequencies of $f = 0.05$ Hz, 0.1 Hz and 0.15 Hz , i.e. 3, 6 and 9 ventilation cycles per minute. The tidal volumes cover a range of 3–10 times of the model dead space which is about 10 ml. As real lungs have a typical dead space of 150 ml and tidal volume during normal breathing is about 500 ml, total liquid ventilation typically covers about 1000 ml (15–20 ml/kg bodyweight) of tidal volume^2^. Hence, the ratio of tidal volume to dead space of our model agrees to physiological conditions. The ventilation frequencies applied here correspond to the frequency range of total liquid ventilation with 3–8 cycles per minute^2,20^ . The peak Reynolds number during the breathing cycle is given by $Re = 4V_T~\rho ~f/D_0\mu$ (at peak velocity in the trachea); the corresponding Womersley number capturing unsteady inertial effects due to oscillatory motion is defined as $\alpha = D_0/2 \sqrt{2 \pi ~f \rho /\mu }$. Here, the values for the density $\rho$ and dynamic viscosity $\mu$ are based on the values of water with $\rho = 1000\,{\mathrm{kg/m}}^3$ and $\mu = 1\cdot 10^{-3}~Pa\cdot s$. All relevant flow parameters are summarized in Table 2. This overview shows that the full range from laminar to turbulent flow conditions as well as typical physiological Womersley numbers are covered here.

**Table 2:** Variation of flow parameters. Velocity, Reynolds numbers *Re* and Womersley numbers $\alpha$, representing peak values in the 0th generation.

f (Hz)	$V_T$ (ml)	$u_{max}$ (m/s)	Re$_0$	$\alpha$
0.05	30	0.054	572	2.9
0.1	30	0.109	1145	4.2
0.15	30	0.164	1717	5.1
0.05	40	0.073	673	2.9
0.05	50	0.091	954	2.9
0.05	60	0.109	1145	2.9
0.1	60	0.219	2290	4.2
0.15	60	0.328	3435	5.1
0.05	80	0.146	1526	2.9
0.05	100	0.182	1908	2.9

### Oxygen concentration measurements

The measurements of the dissolved oxygen concentration are based on the oxygen quenching characteristics of the metallic complex Dichlorotris(1,10)-(phenanthroline)ruthenium(II) ([Ru(phen)$_3$]$^{2+}$). The complex is solved in ethanol (0.4 g:400 ml) and further mixed with 3.6 l of water. This final water-ethanol-dye solution is used as the working fluid during the experiments and also acts as a sensor liquid at the same time. As we showed in a previous work^16^, there are just little differences between using water or an actual PFC liquid in the found concentration maps. The local oxygen concentration is then visualized by the local fluorescence intensity of the working fluid. A higher oxygen concentration is thus associated with a lower fluorescence intensity. The field of view is imaged with a charged coupled device (CCD) camera (monochrome, resolution after pixel binning: 800 $\times$ 150 px, exposure time: $500 \,{\upmu \hbox {s}}$, pco.1600). Illumination is provided by a pulsed high-power LED ($\lambda = {455}$ nm, pulse-length: ${450}\,{\upmu \hbox {s}}$, PT-120, Luminus). A volumetric illumination is applied here. That means, the camera, in top view, acquires an integral image of the fluorescence intensity and not values of the cross sectional mid plane. The excitation wavelengths of the LED are separated from the emission wavelengths of the sensor liquid by an optical high-pass filter ($\lambda _{cut} = {550}$ nm) placed in front of the camera’s CCD chip.

A calibration of the sensor liquid is necessary in order to convert the measured intensity distributions of recorded images into physical oxygen concentrations via the Stern–Volmer equation.

1 $$\begin{aligned} \frac{I_{\mathrm{ref}}}{I} = K_{\mathrm{SV}} \cdot [O_2] + 1 \end{aligned}$$

The calibration is performed prior to the experiments by filling the model with the sensor liquid at ten known oxygen concentrations. The reference probe used here is a dissolved oxygen meter (GMH 3611, Greisinger). The Stern–Volmer constant is then determined by a linear regression of the known intensity-concentration correspondences. The calibration yields a Stern–Volmer constant of $K_{\mathrm{SV}} = 0.1127 \pm 0.002$ l/mg at a temperature of 18.5 $^\circ$C.

In order to model the effect of inspiration and expiration, the liquid in one of the storage containers is enriched with oxygen, while the liquid in the other one is degassed by stripping with nitrogen. The dissolved oxygen concentrations are adjusted to stay at approximately 1 mg/l in the low oxygen concentration tank and at around 8–9 mg/l in the oxygen enriched tank. The liquid’s temperature was constant at 19 ± 0.5 $^\circ$C. Due to the maximum liquid storage volume of the tanks with 4 l, the number of periodic cycles is limited and depends on the investigated tidal volume. Thus, we can record between ten and twenty full breathing cycles. Both, camera and LED are triggered at pre-defined positions of the linear actuator, which enables us to perform phased-locked recordings. The presented results of these measurements are all based on the phase averaging over the total number of recorded breathing cycles. Relative uncertainties of the phase averaged concentrations were found to be in the order of 0.01–0.7%. Altogether, 32 phase positions were measured here.

## Results and discussion

### Qualitative concentration distribution

**Figure 2:**
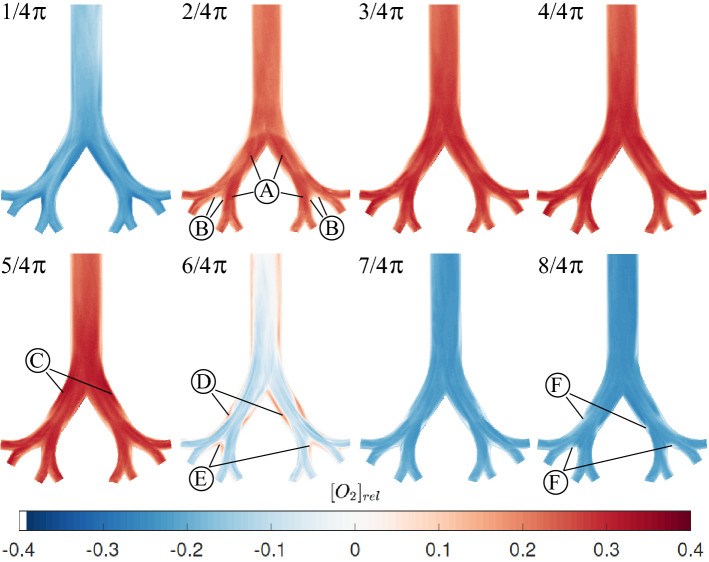
Time-series of the phase averaged, relative oxygen concentration at selected phase angles during one cycle at a tidal volume of 60 ml and a frequency of 0.05 Hz ($Re_0 = 1145$).

At first, we present qualitative concentration visualization results with the aim of identifying important mass transport characteristics. The results are shown for an oscillation frequency of $f = 0.05 \,\hbox {Hz}$ and a tidal volume of 60 ml. Eight phase positions during the whole breathing cycle are included in Fig. 2. The upper part represents the inhalation cycle from the acceleration ($\varphi = 1/4\pi$) until the flow reversal phase ($\varphi = 4/4\pi$) and the lower part shows the expiration phases, again from acceleration ($\varphi = 5/4\pi$) till flow reversal from expiration to inspiration ($\varphi = 8/4\pi$). The images are color coded with respect to the oxygen concentration in relation the cycle’s average. Hence, blue colors denote oxygen poor region, i.e. values below the average, red denotes oxygen rich flow exceeding the average. Overall, concentration values of about ± 35% of the average are observed.

At the beginning of inspiration ($\varphi = 1/4\pi$), streaks of higher concentration emerge in the center of both main branches and an almost symmetric decrease occurs towards the pipe walls. At the inner bifurcation walls, in the carina as well as at the inner walls of the lower generation, the concentration remains at minimum values. This concentration distribution is in contrast to typical velocity profiles in symmetric airway bifurcations which typically feature peak velocities near the inner bifurcation walls^17^. This suggests that concentration and velocity distribution are not similar as originally derived analytically by^21^ for curved tubes.

It should be noted though, that at $\varphi = 6/16\pi$ high oxygen concentration regions clearly emerge at the inner bifurcation walls and inner daughter branches (see supplementary material). However, this distribution already homogenizes at the subsequently recorded phase angle of $\varphi = 7/16\pi$.

At $\varphi = 2/4\pi$, which denotes the peak velocity phase during inspiration, the oxygen concentration distribution has completely changed. The concentration in the whole model is about (20–25)% above the average. Maximum $O_2$ concentrations now occur in the main bifurcation, with a slight asymmetry towards the inner walls, and in the inner branches of the second daughter generation ${}{\textcircled {A}}$. Nevertheless, a clear pathway of oxygen transport cannot be observed. The concentration map rather reveals a well mixed flow across all branches with smaller, dispersed zones of lower concentrations. This suggests that secondary motions contribute to a well mixing within the branches as the flow is still well in the laminar regime (see Table 2). Only the inner wall regions of the second daughter generations exhibit prominent low concentration structures ${\textcircled {B}}$. These are in agreement with the stagnation zones of the flow.

During deceleration ($\varphi = 3/4\pi$), the $O_2$ concentration further increases within the whole model without any qualitative change in distribution. The high values persist beyond the flow reversal phase ($\varphi = 4/4\pi$) until the accelerating expiration ($\varphi = 5/4\pi$). The concentration distribution is qualitatively and quantitatively similar for these two phases. Just a slight increase in $O_2$ concentration can be observed at the outer walls of the main bifurcation ${\textcircled {C}}$, despite the already starting expiration phase.

At the peak expiration phase ($\varphi = 6/4\pi$), striped zones of lower concentration emerge and thus closer resemble the velocity profiles^17^ than during inspiration. Clear separation zones also emerge downstream in the parent branch behind each bifurcation ${\textcircled {D}}$ as well as at inner bifurcation walls ${\textcircled {E}}$. Nevertheless, the stripes are not clearly pronounced anymore and subject to cross motion due to the influence of the secondary vortices. For lower tidal volumes however, these separated high and low concentration layers can be more clearly distinguished from each other.

At the deceleration phase ($\varphi = 7/4\pi$) and flow reversal ($\varphi = 8/4\pi$), this homogenization is further pronounced. Only small spots of low concentration remain in the zones described for the phase of $\varphi = 6/4\pi$ ${\textcircled {F}}$.

### Spatial analysis of oxygen concentrations

**Figure 3:**
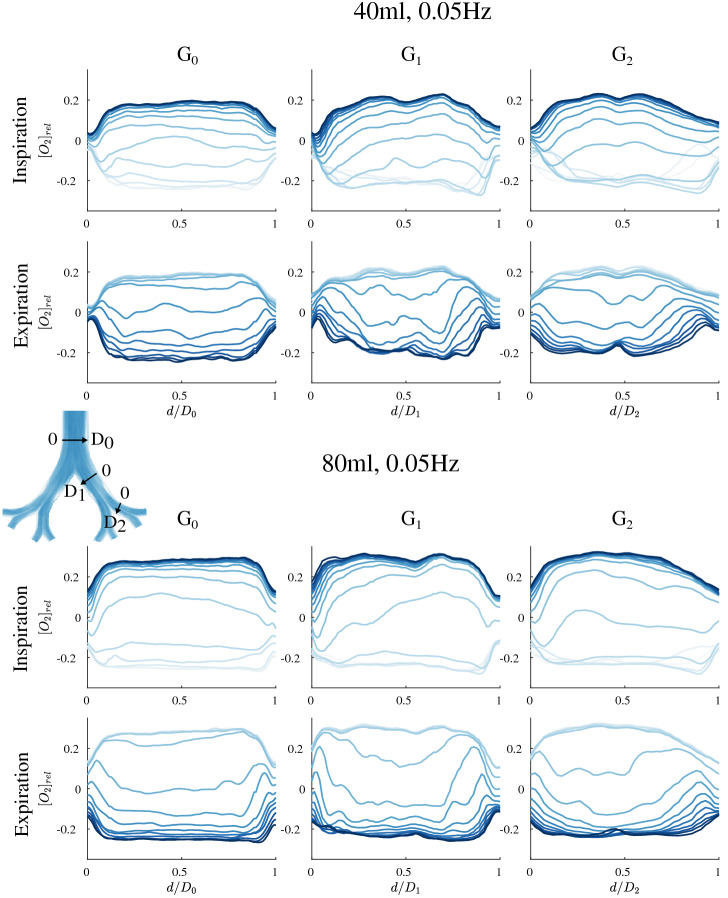
Dissolved oxygen concentration profiles across G$_{\mathrm{0}}$ at $f = 0.05$ Hz and $V_{\mathrm{t}} = 40$ ml (top) and $V_{\mathrm{t}} = 80$ ml (bottom). Each top row is showing the development of the profiles during the inspiration phase ($\varphi = 1/16\pi$–$16/16\pi$) and the bottom rows show the profiles during expiration ($\varphi = 17/16 \pi$–$32/16\pi$), including 16 profiles each. Light and dark blue indicate the beginning and end of the phase, respectively.

Dissolved oxygen concentration profiles within three cross sections in generations $G_0$, $G_1$ and $G_2$ are shown in Fig. 3 for 40 and 80 ml. Since relative changes for different tidal volumes and frequencies are of interest here, the phase averaged oxygen concentration in relation to the cycle’s average is plotted against the normalized profile length for both, the inspirational and expirational phase.

In general, it is recognizable, that with a larger tidal volume, the peak values differ stronger from the phase average. In the following, the oxygen concentration distribution is discussed for the different cross sections.

The concentration profiles in $G_0$ are rather flat for both flow rates and different phase angles. Only the near wall regions till a distance of 0.15 *D* are characterized by a strong concentration gradient. In the first daughter generation ($G_1$) the concentration distribution varies stronger within the cross sections for both flow rates. Moreover, for $V_{\mathrm{t}} = 40$ ml the oxygen concentration in the near wall regions drops further at the beginning of inspiration till $\varphi = 1/4\pi$ since oxygen enriched liquid has obviously not reached this region, yet. This reveals a phase lag between concentrations in central and near wall regions.

For $V_{\mathrm{t}} = 80$ ml, such a drop in oxygen concentration was not observed during inspiration. However, the increase in oxygen concentration is not as symmetric as for $V_{\mathrm{t}} = 40$ ml. While the concentration remains low at the outer bifurcation zone, i.e. the region of recirculations, during the acceleration phase, the concentration at the inner bifurcation suddenly increases between $\varphi = 5/16\pi$–$6/16 \pi$. Within a short time, the peak concentration is reached in the whole cross section and does not change until the expiration phase starts. During expiration, the concentration drops first in the pipe center, with a phase lag of the near wall regions for both flow rates. The location of early change in concentration is in accordance with the peak velocity which also occurs at the same location, i.e. in the pipe center during expiration, in that lung model^17^. This agreement between peak velocity and early change in concentration also confirms the theoretical assumptions from^21^.

In the second generation ($G_2$), the oxygen concentration near the walls even rises between $\varphi = 1/16\pi$ and $\varphi = 2/16\pi$ before it drops to a minimum at $\varphi = 5/16\pi$ and then starts rising again during further inspiration. This behavior indicates an enhanced phase lag between central and near wall concentrations in the lower generations. However, this trend is less pronounced for higher tidal volumes. Overall, the peak concentrations are lower than in $G_0$ and $G_1$ for both tidal volumes, $V_{\mathrm{t}} = 40$ ml and 80 ml.

### Time resolved oxygen concentrations

**Figure 4:**
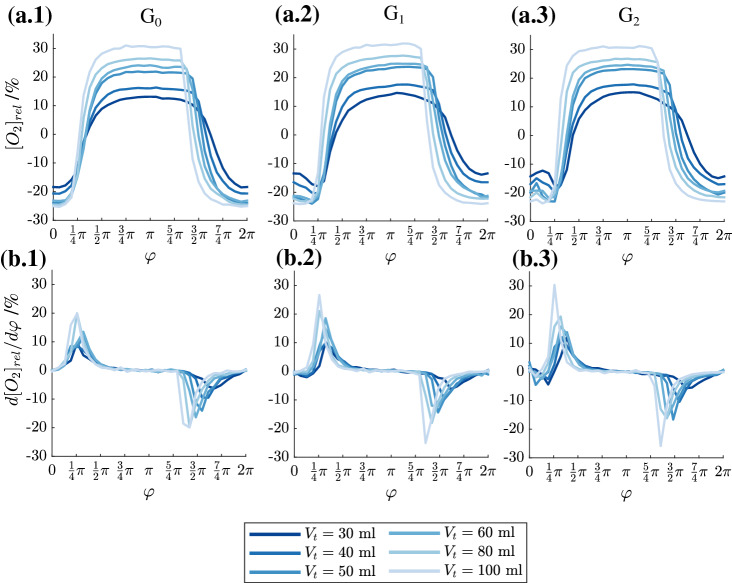
Cross sectional averaged oxygen concentration (**a.1**)–(**a.3**) during a whole breathing cycle and oxygen concentration gradient (**b.1**)–(**b.3**) at $f = 0.05$ Hz and different tidal volumes in different cross section: (1) G$_{\mathrm{0}}$, (2) G$_{\mathrm{1}}$, (3) G$_{\mathrm{2}}$.

The spatially resolved concentrations are now averaged over the cross sections $G_0$–$G_2$ (compare Fig. 3) and their variation for increasing tidal volume from 30 ml to 100 ml during one breathing cycle is shown in Fig. 4a.1–a.3. The frequency was kept constant at f = 0.05 Hz. All subfigures a.x present the relative change of oxygen concentration.

After staying at a low level at the beginning of the inhalation phase, a steep increase in oxygen concentration occurs for all tidal volumes and in all generations. As the concentration gradients (all subfigures b.x) confirm, the concentrations in $G_0$ increase almost at the same phase position of $\varphi = 1/4~\pi$ (Fig. 4b.1) for all tidal volumes. However, during exhalation, an increasing phase lag of $O_2$ concentration occurs with increasing tidal volume and reaches a maximum value of $3/8~\pi$ between 30 ml and 100 ml.

A comparison of the onset of concentration increase between the generations $G_0$–$G_2$ reveals, that small phase lags already occur during inspiration with a maximum of $1/8~\pi$ between 30 ml and 100 ml (Fig. 4b.2,b.3). During exhalation, the phase lag is again similar to the behavior in $G_0$, i.e., three times of that during inhalation. These phase lags between low and high tidal volumes at the same frequency originate from higher flow rates for higher tidal volumes which thus cause similar liquid volumes to reach the lung model generations earlier within the breathing cycle.

Nevertheless, the phase lag for different tidal volumes does not increase further in deeper generations. That means the temporal behavior does obviously not depend on the distance of the generation from the model entrance and more general, phase lags do not depend on the model dead space.

**Figure 5:**
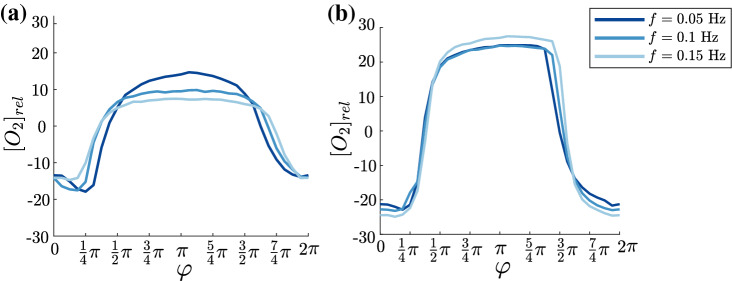
Cross sectional averaged oxygen concentration at constant tidal volume of 30 ml (**a**) and 60 ml (**b**) for different frequencies.

However, the cross sectional mean concentration magnitudes strongly depend on the tidal volume. For increasing tidal volume higher deviations from the averaged oxygen concentration occur. This trend appears to be almost linear. For inspiration, a doubling in tidal volume leads approximately to a twofold increase in oxygen concentration. During expiration, these variations are smaller with $-\,15$% at 30 ml and $-\,25$% at 100 ml. Note here again , that all tidal volumes exceed the dead space of the lung model and the peripheral piping. Moreover, increasing the supplying pipe lengths at the model entrance and exits did not change the temporal and spatial concentration distributions. Hence, these differences cannot be explained by unreachable generations for oxygen rich/poor liquid within one cycle. One explanation could be higher flow velocities which also induce stronger secondary flows and thus lateral mixing. In order to verify this assumption, the flow frequency is increased to 0.1 Hz and 0.15 Hz while keeping the tidal volume constant at 30 ml and 60 ml, respectively. The temporal variation of the averaged concentration in cross section $G_1$ for these two different tidal volumes and increasing frequencies is shown in Fig. 5.

However, as can be seen in Fig. 5, the differences for increasing frequency are rather low. Moreover, for low tidal volume of only 30 ml (Fig. 5a) peak values of averaged concentrations even decrease slightly with increasing frequency. For higher tidal volume (Fig. 5b) the concentration profiles for all frequencies reveal a very similar shape, now with the maximum concentration change for the highest frequency. Hence, an enhanced lateral mixing due to higher velocities does not influence oxygen concentrations in the cross sections. The change from laminar to turbulent regime which applies for 60 ml from 0.1 to 0.15 Hz does obviously have only neglectable influence on concentration distribution over time.

## Summary and conclusion

Here, we successfully visualized oxygen transport in a simplified in vitro model of the human respiratory airways by the method of fluorescence quenching. The concentration distribution could be investigated with high temporal and spatial resolution. To the best of the authors knowledge, this is the first time that the temporal and spatial oxygen concentration distribution could be visualized in a lung model. In patients, the conductive and respiratory airways are still a black box and only oxygen concentration in the inhaled air and blood gas concentration can be measured. The evaluation of oxygen concentration in the complete model revealed that there are only slight similarities with velocity patterns known from literature in similar models. Comparable structures disclose only short living times and are subject to fast homogenization due to lateral mixing, already before the onset of turbulence. The increase of oxygen concentration in the lung model does not occur linearly but follows an exponential rise during the accelerating inhalation phase. This increase and also decrease occurs earlier in phase for higher volumes, although downstream regions with higher distance to inlets are not subject to any phase shifts. Only radial phase shift and concentration gradients could be observed. The results for all tidal volumes exhibit a constant maximum oxygen concentration level throughout the model. This constant high level persists during half of the complete cycle time. However, there is a strong dependency on the tidal volume. Although all tidal volumes exceed the model’s dead space, considerably higher concentration levels are reached for higher tidal volumes. Since increasing the length of the feeding pipe in front the inlet did not even have an influence on temporal and spatial oxygen distribution, one can conclude that the airway dead space has only neglectable influence on peak concentrations.

Although the measurement technique allows new insight into oxygen transport in human airway models, the model as well as the technique feature some limitations. It is expected that in a 3D model, clear distinguishable stripes as in the planar model might not occur due to enhanced secondary motions generated by the 3D geometry structure. The influence of further bifurcating branches and additional generations on $O_2$ concentration distribution in deeper branches could moreover not be investigated here. The integral measurement of the fluorescence intensity and thus $O_2$ concentration might deviate from mid plane measurements and could thus closer resemble velocity measurements in the central plane.

## Supplementary Information

Supplementary Video 1.

Supplementary Legends.

## References

[1] Klystra JA, Tissing MO, Ban der Maen A (1962). Of mice as fish. ASAIO J..

[2] Greenspan JS, Wolfson MR, Shaffer TH (2000). Liquid ventilation. Semin. Perinatol..

[3] Hirschl RB, Conrad S, Kaiser R, Zwischenberger JB, Bartlett RH, Frank B, Cardenas V (1998). Partial liquid ventilation in adult patients with ARDS. Ann. Surg..

[4] Galvin IM, Steel A, Pinto R, Ferguson N, Davies M (2013). Partial liquid ventilation for preventing death and morbidity in adults with acute lung injury and acute respiratory distress syndrome (Review). Cochrane Database Syst. Rev..

[5] Chenoune M, Lidouren F, Adam C, Pons S, Darbera L, Bruneval P, Ghaleh B, Zini R, Dubois-Randé JL, Carli P, Vivien B, Ricard JD, Berdeaux A, Tissier R (2011). Ultrafast and whole-body cooling with total liquid ventilation induces favorable neurological and cardiac outcomes after cardiac arrest in rabbits. Circulation.

[6] Nadeau M, Denaclara JY, Tissier R, Walti H, Micheau P (2018). Patient-specific optimal cooling power command for hypothermia induction by liquid ventilation. Control Eng. Pract..

[7] Rambaud J, Lidouren F, Sage M, Kohlhauer M, Nadeau M, Fortin-Pellerin É, Micheau P, Zilberstein L, Mongardon N, Ricard JD, Terada M, Bruneval P, Berdeaux A, Ghaleh B, Walti H, Tissier R (2018). Hypothermic total liquid ventilation after experimental aspiration-associated acute respiratory distress syndrome. Ann. Intensive Care.

[8] Wei F, Wen S, Wu H, Ma L, Huang Y, Yang L (2019). Partial liquid ventilation-induced mild hypothermia improves the lung function and alleviates the inflammatory response during acute respiratory distress syndrome in canines. Biomed. Pharmacother..

[9] Kohlhauer M (2020). A new paradigm for lung-conservative total liquid ventilation. EBioMedicine.

[10] Halpern, D., Fujioka, H., Takayama, S. & Grotberg, J. B. NIH Public Access. *Respir. Physiol. Neurobiol.* **163**, 222–231 (2008). 10.1016/j.resp.2008.05.012 PMC2592688 18585985

[11] Quintel M, Hirschl RB, Roth H, Loose R, Van Ackern K (1998). Computer tomographic assessment of perfluorocarbon and gas distribution during partial liquid ventilation for acute respiratory failure. Am. J. Respir. Crit. Care Med..

[12] Laukemper-Ostendorf S, Scholz A, Buerger K, Heussel CP, Schmittner M, Weiler N, Markstaller K, Eberle B, Kauczor H-U, Quintel M, Thelen M, Schreiber WG (2002). $$^19$$F-MRI of perflubron for measurement of oxygen partial pressure in porcine lungs during partial liquid ventilation. Magn. Reson. Med..

[13] Schnabel C, Gaertner M, Kirsten L, Meissner S, Koch E (2013). Total liquid ventilation: A new approach to improve 3D OCT image quality of alveolar structures in lung tissue. Opt. Express.

[14] Sage M, Stowe S, Adler A, Forand-Choinière C, Nadeau M, Berger C, Marouan S, Micheau P, Tissier R, Praud J-P, Fortin-Pellerin É (2018). Perflubron distribution during transition from gas to total liquid ventilation. Front. Physiol..

[15] Janke T, Bauer K (2017). Visualizing dissolved oxygen transport for liquid ventilation in an in vitro model of the human airways. Meas. Sci. Technol..

[16] Janke, T., Schwarze, R. & Bauer, K. Qualitative in vitro measurements of dissolved oxygen concentrations in perfluorocarbon during liquid ventilation. In *Proc. 18th Int. Symp. Flow Vis.* (2018).

[17] Bauer K, Nof E, Sznitman J (2019). Revisiting high-frequency oscillatory ventilation in vitro and in silico in neonatal conductive airways. Clin. Biomech..

[18] Janke T, Schwarze R, Bauer K (2017). Measuring three-dimensional flow structures in the conductive airways using 3D-PTV. Exp. Fluids.

[19] Adler K, Brücker C (2007). Dynamic flow in a realistic model of the upper human lung airways. Exp. Fluids.

[20] Tawfic QA, Kausalya R (2011). Liquid ventilation. Oman Med. J..

[21] Eckmann DM, Grotberg JB (1988). Oscillatory flow and mass transport in a curved tube. J. Fluid Mech..

