# Supplementary material for: Oxygen transport during liquid ventilation: an in vitro study
Source: Sci Rep. 2022 Jan 24;12:1244. doi: 10.1038/s41598-022-05105-1 (PMC8786849; doi:10.1038/s41598-022-05105-1)
Supplement: Supplementary file 2 — Supplementary Legends. [file 41598_2022_5105_MOESM2_ESM.pdf]

The supplementary video shows concentration maps of all recorded phase angles for a tidal volume of V_T = 60 ml and a frequency of f = 0.05 Hz (Re = 1145). Values are given as relative oxygen concentration. The video starts at the flow reversal phase from expiration to inspiration.
